# hMSCs in contact with DMSO for cryopreservation: Experiments and modeling of osmotic injury and cytotoxic effect

**DOI:** 10.1002/bit.28174

**Published:** 2022-07-28

**Authors:** Gabriele Traversari, Francesco Delogu, Santiago Aparicio, Alberto Cincotti

**Affiliations:** ^1^ Dipartimento di Ingegneria Meccanica, Chimica e dei Materiali Università degli Studi di Cagliari Cagliari Italy; ^2^ Department of Chemistry University of Burgos Burgos Spain

**Keywords:** cryopreservation, cytotoxicity, DMSO, experiment, modeling, osmotic injury

## Abstract

In this study a combined analysis of osmotic injury and cytotoxic effect useful for the optimization of the cryopreservation process of a cell suspension is carried out. The case of human Mesenchymal Stem Cells (hMSCs) from Umbilical Cord Blood (UCB) in contact with dimethyl sulfoxide (DMSO) acting as Cryo‐Protectant Agent (CPA) is investigated from the experimental as well as the theoretical perspective. The experimental runs are conducted by suspending the cells in hypertonic solutions of DMSO at varying osmolality, system temperature, and contact times; then, at room temperature, cells are pelleted by centrifugation and suspended back to isotonic conditions. Eventually, cell count and viability are measured by means of a Coulter counter and flow‐cytometer, respectively. Overall, a decrease in cell count and viability results when DMSO concentration, temperature, and contact time increase. A novel mathematical model is developed and proposed to interpret measured data by dividing the cell population between viable and nonviable cells. The decrease of cell count is ascribed exclusively to the osmotic injury caused by expansion lysis: excessive swelling causes the burst of both viable as well as nonviable cells. On the other hand, the reduction of cell viability is ascribed only to cytotoxicity which gradually transforms viable cells into nonviable ones. A chemical reaction engineering approach is adopted to describe the dynamics of both phenomena: by following the kinetics of two chemical reactions during cell osmosis inside a closed system it is shown that the simultaneous reduction of cell count and viability may be successfully interpreted. The use of the Surface Area Regulation (SAR) model recently proposed by the authors allows one to avoid the setting in advance of fixed cell Osmotic Tolerance Limits (OTLs), as traditionally done in cryopreservation literature to circumvent the mathematical simulation of osmotic injury. Comparisons between experimental data and theoretical simulations are provided: first, a nonlinear regression analysis is performed to evaluate unknown model parameters through a best‐fitting procedure carried out in a sequential fashion; then, the proposed model is validated by full predictions of system behavior measured at operating conditions different from those used during the best‐fit procedure.

## 1 INTRODUCTION

In tissue engineering, the preservation of biological material is a core technology to bring cell‐based products to market on‐demand (Cincotti & Fadda, 2012; Karlsson & Toner, 2000). The principal preservation method consists of freezing the bio‐specimens to cryogenic temperatures to take advantage of the preservative power of cold. In fact, compared to other preservation methods like maintaining biosamples in continuous culturing, cryopreservation has the benefits of affording long shelf lives, genetic stability, reduced microbial contamination risks, and cost‐effectiveness (Karlsson & Toner, 2000). The flip side of this method is that cryopreserved biological material can be damaged by the cryopreservation process itself, with a significant loss of viable or functional cells (Karlsson & Toner, 2000; Malpique et al., 2009; Mazur et al., 1972; Mazur, 2004; Naaldijk et al., 2012). While such loss is acceptable for some cell lineages for research application, it becomes unacceptable in clinical practices, especially those involving human Mesenchymal Stem Cells (hMSCs) from Umbilical Cord Blood (UCB) whose collection and isolation is known to be difficult (Bieback et al., 2004; Casula et al., 2017; Gao et al., 1995). Thus, optimization of the operating conditions adapted to the osmotic behavior of the specific cell lineage under investigation is crucial for cryopreservation (Benson, 2021; Casula et al., 2017, 2019; Cincotti & Fadda, 2012; Fadda et al., 2009, 2010, 2011a, 2011b; Hughes et al., 2012; Karlsson & Toner, 2000).

Cryopreservation of a cell suspension consists of a sequence of different steps, sometimes in combination: osmotic addition of a permeant Cryo‐Protectant Agent (CPA) as dimethyl sulfoxide (DMSO), cooling, storage, thawing, and CPA removal to return to the physiological environment for specific usages. CPAs are chemicals added to the medium containing the cells to prevent damage from ice formation both thermodynamically and kinetically, that is, by depressing the freezing point as well as by increasing the viscosity and glass transition temperature of the solution to favor vitrification over ice formation. With the exception of storage at very low temperatures, all the steps listed above are potentially able to damage the cells due to the physicochemical and biological phenomena involved: osmotic injury due to excessive cell volume excursions, CPA cytotoxicity, and intracellular ice formation during freezing or recrystallization from glass during warming. Unfortunately, the number of experimental variables and parameters involved in a cryopreservation protocol is prohibitively large to permit a rigorous optimization of the process (Benson, 2021; Casula et al., 2019; Karlsson & Toner, 2000): not only cooling and thawing rates need to be optimized to limit the lethal intracellular ice formation or recrystallization but also concentration, temperature, and temporal duration of CPA addition and removal usually carried out in a step‐wise fashion, demand a careful design to limit osmotic injury and cytotoxicity. On the other hand, best practices may be defined through mathematical modeling and numerical simulations (Benson, 2021; Cincotti & Fadda, 2012; Fadda et al., 2009, 2010, 2011a, 2011b; Karlsson & Toner, 1996), provided that the adopted mathematical model is capable to describe properly the behavior of the system under investigation.

Traditionally the problem of optimizing the cryopreservation of a cell suspension has been attacked by analyzing separately the different phenomena involved, both experimentally and theoretically (Benson, 2021; Casula et al., 2019; Fadda et al., 2010; Karlsson & Toner, 2000). In particular, during prefreezing addition and post‐thawing removal of CPAs cells may be damaged only by CPA cytotoxicity and excessive volume excursions driven by osmosis. For the osmotic injury several possible mechanisms have been hypothesized so far (Gao et al., 1995): (1) mechanical rupture of the cell membrane during swelling in hypo‐osmotic conditions, that is, expansion lysis (Wolfe et al., 1986); (2) damage of cell membrane caused by frictional forces between exchanged water and membrane pores (Muldrew & McGann, 1994); (3) cell shrinkage in hyperosmotic condition resisted by cytoskeleton components, with the resultant interaction between shrunken cell membrane and cytoskeleton damaging the cells (Meryman, 1970); (4) irreversible membrane fusion/change induced by cell shrinkage, so that the effective area of cell membrane is reduced and, when returned to isotonic condition, the cells lyse before their normal volume is recovered (Steponkus & Wiest, 1979); (5) a net influx of nonpermeating solutes caused by hyperosmotic stress so that, when cells are returned to isotonic conditions, they swell beyond their normal isotonic volume and lyse (Lovelock, 1953; Mazur et al., 1972). These mechanisms suggested the existence of a safety range for osmotic excursions delimited by a minimum and a maximum cell volume where osmotic injury does not occur, that is, the Osmotic Tolerance Limits (OTLs). The determination of the OTLs has been the subject of several experimental investigations in the cryopreservation literature, even if a mathematical model capable to describe osmotic injury and the corresponding decrease of cell count has never been proposed so far. Since the extent of cell shrinkage or swelling depends on membrane permeabilities to water and permeant solutes, OTLs are expected to vary from cell to cell lineage just like the cytotoxic effect of DMSO.

The latter one, according to some experimental studies, should be related to DMSO interaction with the phospholipid double‐layer composing the cell membrane, capable to reduce membrane thickness (Hughes et al., 2012) or promote the formation of pores (Fernandez & Reigada, 2014). In fact, these mechanisms are confirmed by molecular dynamics simulations which show that cell membrane response to cytotoxicity varies when increasing the concentration level of DMSO (Gurtovenko & Anwar, 2007): first, at relatively low DMSO concentrations (~2.5−7.5 mol%), only a decrease in membrane thickness takes place, while at intermediate concentrations (~10−20 mol%) a transient formation of water pores occurs, and, finally, at higher concentrations (~25−100 mol%) the double‐layer structure of cell membrane is destroyed. However, it is still not clear how DMSO interacts with the internal structures of the cells, which molecular mechanisms are involved, and, most important, how they are related to the reduction of cell viability measured at a larger, macroscopic scale by means of the experimental techniques currently available. For these reasons, nowadays the term cytotoxic effect is still used to refer to global, generic damage (other than osmotic injury) that is experienced by the cells contacted with a toxic CPA, and measured macroscopically by checking cell membrane integrity or viability/functionality through different biological assays.

So far the contact of cells with CPAs has been investigated both experimentally and theoretically by following three different approaches: studying only the injury caused by excessive osmotic excursions, focusing only on the cytotoxic effect, or examining the two phenomena together in a combined analysis. In the first case, osmotic injury is analyzed alone by limiting or neglecting cytotoxicity with the specific aim of determining OTLs, that is, by working at small CPAs concentrations, low temperatures, and short contact times or directly without using any cytotoxic CPA. This way, the OTLs of human spermatozoa (Gao et al., 1995), human oocytes (Mullen et al., 2004; Newton et al., 1999), human red blood cells (Lusianti et al., 2013; Zhurova et al., 2014), and mouse embryonic stem cells (Kashuba et al., 2014) were experimentally determined. In the second approach, tissues instead of cell suspensions are typically addressed: by assuming that the osmotic shifts do not contribute to cell death due to protection of the tissue matrix, cytotoxicity is analyzed alone and osmotic injury is neglected (Elmoazzen et al., 2007). This is the case of articular cartilage from pigs (Elmoazzen et al., 2007) or human dermal tissue (Wang et al., 2007) contacted with hypertonic solutions of DMSO at varying osmolalities, temperatures, and contact times. Last, when aiming to design optimal CPA prefreezing addition and post‐thawing removal the third approach is adopted. The goal is sought by avoiding the osmotic injury while simultaneously limiting the cytotoxic effect, that is, by adopting a multistep strategy for CPA addition/removal while working at low temperatures, CPA concentrations, and short contact times, respectively. This way the case of human CD34^+^ stem cells from UCB with DMSO (Hunt et al., 2003a, 2003b), human and murine oocytes, and adherent endothelial cells with DMSO, propylene, or ethylene glycol (Benson et al., 2012; Davidson et al., 2014, 2015; Karlsson et al., 2014) were investigated.

In all these studies the classic Kedem and Katchalsky (1958) or two‐parameter formalisms (Kleinhans, 1998) were used for the theoretical description of cell volume excursions during osmosis, even though these mathematical models are not capable to predict osmotic injury. To overcome this problem, the simulation of the osmotic damage was circumvented by confining cell volume excursions within the safety range of preset OTLs, experimentally determined in advance. Typically, the OTLs were identified as fixed values of the isotonic volume of a given cell line, whereas it was experimentally demonstrated that rupture of plasma membrane depends on the rate of osmotic expansion which causes an excessive but temporary increase of the membrane surface tension (Wolfe et al., 1985, 1986). This means that OTLs are not constant but actually vary for any given cell line, depending on the adopted operating conditions like temperature and the osmotic driving force; according to (Lemetais et al., 2012; Wolfe et al., 1985, 1986) the rupture of plasma membrane should be considered a stochastic phenomenon affecting a variable number of cells but allowing others to survive the expansion.

Moreover, as originally pointed out back in the 50 s (Lovelock, 1953), lysis during swelling is generally thought to be more lethal than excessive shrinkage (Gao et al., 1995; Hunt et al., 2003a; Kashuba et al., 2014; Newton et al., 1999; Zhurova et al., 2014), and osmotic injury is found especially damaging only when returning back to isotonic conditions after suspending the cells in hypertonic solutions (Gao et al., 1995; Lusianti et al., 2013).

Therefore, assuming that expansion lysis rather than excessive shrinkage is the true cause of osmotic damage, in this study a combined analysis of cytotoxicity and osmotic injury is carried out. Experimentally, cell count and viability are measured through a Coulter counter and flow‐cytometer, respectively, only after hMSCs from UCB have been first contacted with hypertonic solutions of DMSO, pelleted by centrifugation and then suspended back to isotonic conditions. Theoretically, the decrease of cell count and viability measured at increasing osmolality, temperature and contact times are ascribed to expansion lysis and cytotoxicity, respectively, by means of a novel mathematical model based on prime principles of conservation: the two phenomena are described as two chemical reactions evolving in a closed system whose reactants/products are the viable and nonviable cells of the population subjected to osmotic excursions. Cell osmosis is described by the Surface Area Regulation (SAR) model, where the temporary variations of cell membrane tension are accounted for: if an excessive, rapid swelling occurs both viable and nonviable cells burst in a stochastic way and are transformed into debris, thus accounting for cell count decrease. On the other hand, the decrease of cell viability due to cytotoxicity is depicted as the gradual transformation of viable cells into nonviable ones catalyzed by the DMSO accumulated inside the cells during osmosis.

Comparisons between experimental data and theoretical simulations are provided: first, a nonlinear regression analysis is performed to evaluate unknown model parameters through a best‐fitting procedure conducted in a sequential fashion; then, the proposed model is validated by full predictions of system behavior measured at operating conditions different from those used during the best‐fit procedure. This way setting in advance fixed OTLs as traditionally done in cryopreservation literature to circumvent the mathematical simulation of osmotic injury is no longer required: the proposed model is capable to describe cell behavior outside the safety range delimited by the OTLs.

## 2 EXPERIMENTAL SECTION

### 2.1 Experimental setup and operating conditions

**Figure 1:**
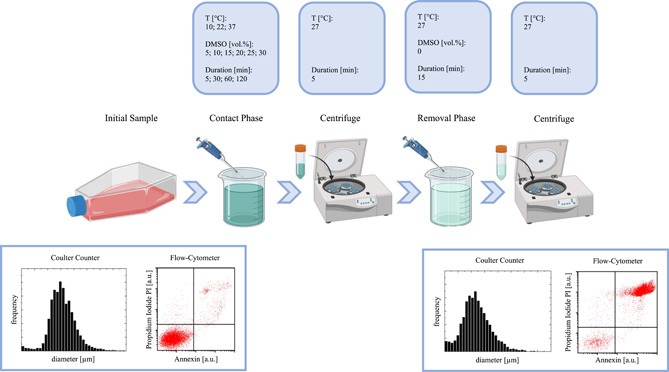
Experimental protocol and operating conditions

The hMSCs from UCB of three different donors were collected and isolated as described in (Casula et al., 2017). The experimental runs carried out in this study consist in two sequential stages: the contact stage of the cells with DMSO when the permeant CPA is loaded into the cytoplasm, followed by removal when the CPA exits the cells. Both stages are carried out in a single step. Between the two stages, cells are pelleted by centrifugation (Figure 1).

In particular, the contact stage is performed by suspending about 10^6^ cells in 1 ml of hypertonic solutions containing DMSO (Sigma‐Aldrich) added to isotonic phosphate‐buffered saline (PBS) at varying osmolality, temperature, and duration as shown in Table 1 and Figure 1. More specifically, system response at seven DMSO concentrations (from 0 to 30 vol.%), three temperatures (from 10°C to 37°C) and four contact times (from 5 to 120 min) are investigated. It is worth noting that, the concentration range explored in this study is below 10 mol% of DMSO, as reported in the footnote of Table 1: according to (Gurtovenko & Anwar, 2007) in these experimental runs the integrity of the cell membrane is not completely damaged by the cytotoxic effect and its water permeability is not affected and remains constant as no transient formation of water pores is actually taking place at these relatively low DMSO concentration levels. The cases of the lowest temperature (i.e., 10°C) combined with the shortest contact times (i.e., 5 and 30 min), as well as the highest temperature (i.e., 37°C) with the longest contact time (i.e., 120 min) are skipped on purpose, to avoid the negligible or excessive cell damage expected at these extreme conditions, respectively. A freezing‐point‐depression osmometer (Advanced Micro Osmometer Model 3300; Advanced Instruments) is used to measure solution osmolality. The system temperature is controlled within a ±0.2°C interval by circulating water‐NaCl bath at 2% wt./vol. as suggested by (Lee et al., 2004). To this aim, isotonic cells were injected into a suitably manufactured beaker allowing perfect mixing and dispersion in the suspending solution.

**Table 1:** Operating conditions for the contact stage with DMSO

DMSO [vol.%]	Contact temperature [°C]	Contact time [min]			
0; 5; 10; 15; 20; 25; 30^a^	10			60	120
0; 5; 10; 15; 20; 25; 30	22		30		120
0; 5; 10; 15; 20; 25; 30	37	5	30	60	

*Note*: The following removal stage is always performed by suspending the cells back into isotonic PBS at 27°C for 15 min, after centrifugation for 5 min at 400*g*.

Abbreviation: DMSO, dimethyl sulfoxide.

^a^ 5; 10; 15; 20; 25; 30 vol.% of DMSO correspond to about 705; 1410; 2110; 2820; 3520; 4225 mOsm/L or 1.3; 2.5; 3.8; 5.1; 6.3; 7.6 mol%.

The subsequent removal stage is performed by suspending the cells back into isotonic PBS in a single step at 27°C for 15 min, after centrifugation at 400*g* for 5 min. Cell count and viability are measured by means of a Coulter counter and a flow‐cytometer, respectively, only initially (before the contact stage) and finally (at the end of the removal stage). Separate experimental runs are performed for individual donors and repeated at least three times, before pooling and averaging data.

### 2.2 Cell count

**Figure 2:**
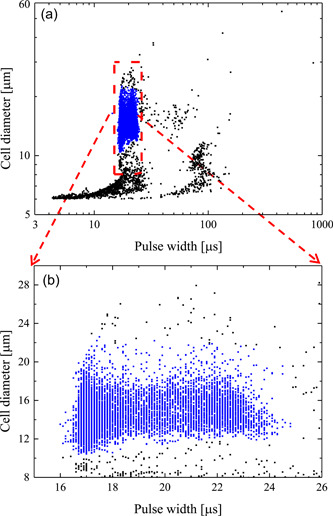
Data treatment for Coulter counter measurements to filter out cells from debris and agglomerates: Gating of a representative diameter versus pulse width dot‐plot (a); zooming in the gated region (b).

Cell count is measured using a Coulter Counter Multisizer 4 (Beckman Coulter), initially calibrated using latex beads (diameter 10 μm, Beckman Coulter). Before each experiment, the instrument electrolyte solution was replaced by the appropriate hypertonic solution to avoid a mismatch with the sample solution and consequent electrical conductivity gradients (Bryan et al., 2012). Coulter Counter's capability of measuring the number of cells of a relatively large population represents a significant advantage over micrographic analysis and direct microscopic inspection, which are necessarily restricted to small samples. In this regard, Coulter Counter's performances are comparable with those of flow‐cytometers (Acker et al., 1999). However, since impedance measurements do not discriminate single cells by debris or cell agglomerates, a data treatment is required. To this aim, the dot plot measured by the Coulter counter is adopted, as shown in Figure 2 where a representative case is reported. Here individual points correspond to the amplitude and duration of any single event (i.e., voltage pulse with height and width) detected by the Coulter counter during its passage through the sensing zone. In particular, the pulse amplitude is related to cell size, and, after calibration, can be expressed as cell diameter measured in µm. Debris and cell agglomerates are filtered out through gating as shown in Figure 2, by considering only the events falling within the red rectangular representing single, intact cells, that is, by assuming that debris and cell agglomerates are characterized by relatively small diameters and large width, respectively.

### 2.3 Cell viability

Cell viability is determined by a dual staining assay analyzed in a flow cytometer (FACScan Becton Dickinson), to separate viable cells from apoptotic and necrotic ones. After the removal stage, and centrifugation at 400*g* for 5 min, the cells are suspended in X1 Binding Buffer at a concentration of 10^6^ cells/ml. Later 100 μl of the resulting solution are transferred to a 5 ml culture tube, where 5 μl of Alexa Fluor® 488 Annexin V and 5 μl of Propidium Iodide (PI) (Life Technologies) is added for staining. Then, after incubation for 15 min at 27°C, 400 μl of X1 Binding Buffer are added and the cells are eventually analyzed in the flow‐cytometer by examining a constant number of events (i.e., 10^4^).

**Figure 3:**
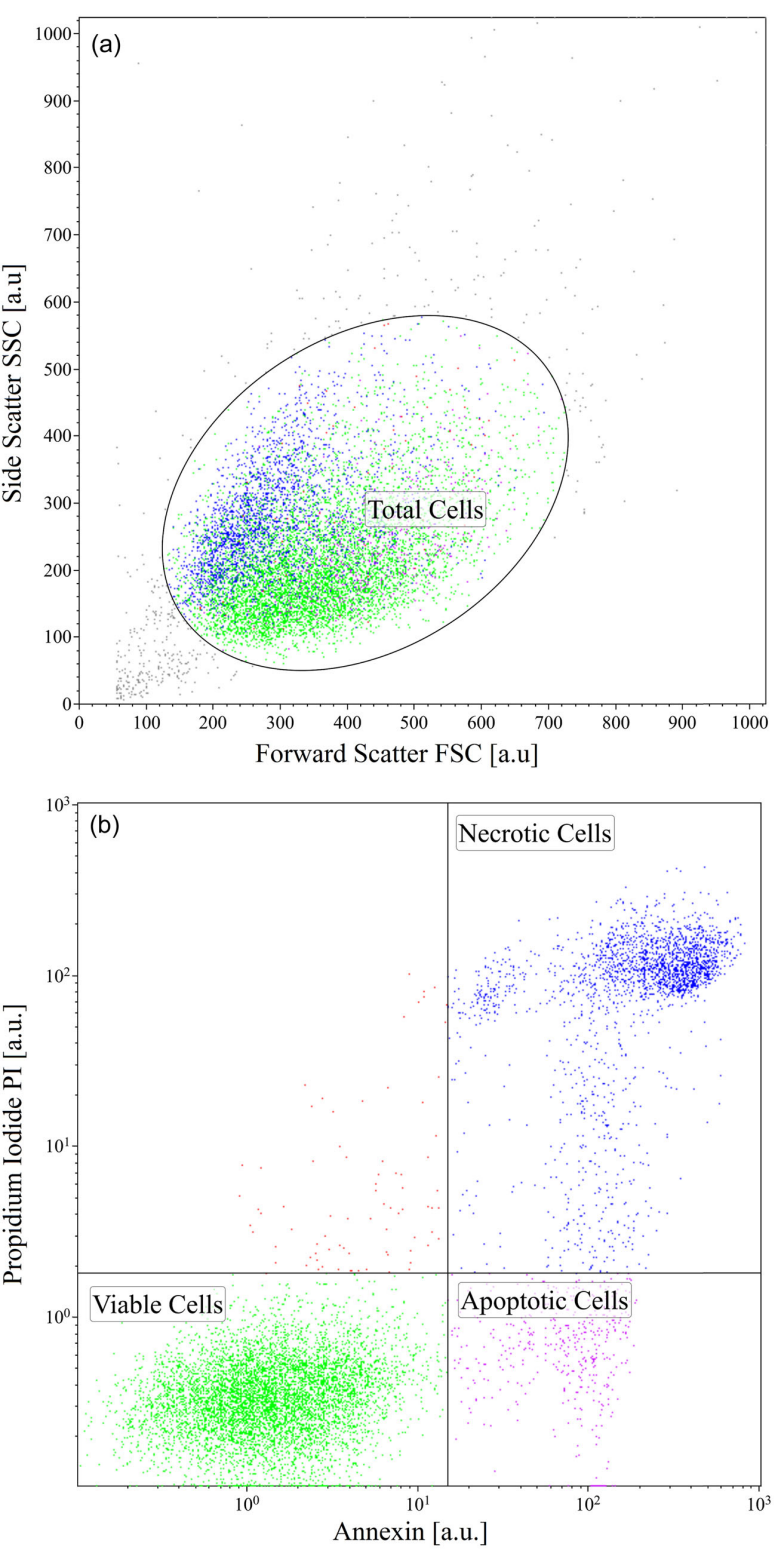
Data treatment for flow‐cytometer by means of software Kaluza: dot plot of SSC versus FSC with elliptical gating to isolate cells from debris and agglomerates (a); dot plot of Propidium Iodide versus Annexin expressions to separate viable cells from apoptotic and necrotic ones (b). FSC, Forward Scatter; SSC, Side Scatter.

Data from the flow‐cytometer need to be treated as well. By means of Kaluza software (Beckman Coulter), first debris and cell agglomerates are filtered out through an elliptical gating on the dot‐plot of Side Scatter (SSC) versus Forward Scatter (FSC) measured by the flow‐cytometer as shown in Figure 3a, where a representative case is reported. Here individual points correspond to FSC (i.e., size) and SSC (i.e., internal complexity) for any single event detected by the flow cytometer. Then, on the selected population of cells, the dot plot of PI versus Annexin expressions is analyzed to differentiate viable cells from apoptotic and necrotic ones, as shown in Figure 3b, where cells fall within four different quadrants are identified. In fact, apoptotic phenomena are characterized by morphological changes that appear in a sequential fashion, including loss of asymmetry and attachment in the plasma membrane, condensation of the cytoplasm and nucleus, and internucleosomal cleavage of DNA. Loss of plasma membrane is one of the earliest changes. In apoptotic cells, the Phospholipid phosphatidylSerine (PS) in the membrane is translocated from the inner to the outer leaflet of the plasma membrane, thereby exposing PS to the external cellular environment. On the other hand, necrotic cells simply show a damaged membrane. Annexin is a 35−36 kDa Ca^2+^ dependent phospholipid‐binding protein, that shows high affinity for PS. Since Annexin is a nonpermeable protein, it binds to cells only when PS is exposed in the outer layer. Conjugating Annexin V with fluorochromes as Fluorescein IsoThioCyanate allows detecting early apoptotic cells through flow cytometric analysis. The loss of membrane integrity typical of necrotic processes is detected by a vital dye such as PI: viable cells with intact membranes exclude PI, whereas membranes of dead and damaged cells are permeable to PI, a DNA intercalant. Both negative and positive controls are performed. In particular, a negative control is performed by analyzing nontreated cells (i.e., the initial isotonic cells), while positive control is performed by analyzing treated cells (i.e., cells subjected to the worst conditions after contact and removal phase with DMSO to achieve zero viability). Besides, to set properly the working parameters of the flow‐cytometer, auto‐fluorescence is checked too as a negative control on unstained cells.

Therefore, on the basis of the dot plot for cells positive or negative to Annexin and PI shown in Figure 3b, cells may be divided according to three different conditions: viable cells (negative to both Annexin and PI staining); apoptotic cells (positive to Annexin but negative to PI staining); necrotic cells (positive to both Annexin and PI staining). The case of cells positive to PI but negative to Annexin staining is not taken into account, since a cell without membrane integrity cannot be negative to Annexin marker, in principle. As apparent in Figure 3b, the number of these cells is negligible, accordingly.

Viability is finally determined as the ratio between the number of viable cells and the total number of cells shown in Figure 3b. More specifically, the total number of cells in Figure 3b is obtained by summing up the viable, necrotic, and apoptotic cells and is equal to the number of cells resulting from the gating in Figure 3a. It is worth noting that, the total number of cells measured by the flow‐cytometer is assumed to be proportional to the total number of cells measured by the Coulter counter as they are both determined after the removal of debris and cell agglomerates. On the other hand, these two numbers do not coincide since they are independently measured by two different apparatuses working at two different conditions: the Coulter counter measures the number of cells suspended in a preset volume of solution, while the flow‐cytometer measures the distribution of viable, necrotic, and apoptotic cells within a preset number of cells.

## 3 MODELING SECTION

The osmotic behavior of hMSCs from UCB is described by means of the SAR model (Casula et al., 2019; Traversari & Cincotti, 2021), whose Equations are reported in Tables A1−A2 of the Appendix section. For the sake of brevity, a brief description of this model is provided only there. Here it is worth noting that the SAR model was developed and validated to interpret the peculiar osmotic behavior of these cells measured when contacting with DMSO at relatively low osmolalities and short contact times, that is, when both osmotic injury and cytotoxicity do not occur. This way, the value of the adjustable parameters of the SAR model was determined as reported in Table A3 of the Appendix section. In particular, water, DMSO, and Ion/salt permeabilities to hMSCs membrane are given with their Arrhenius‐like dependence, that is, $L_{P}=L_{P}^{\infty }\;\cdot \;\text{exp}\left(-\frac{E_{a,W}}{\mathrm{RT}}\right)$; $P_{\text{CPA}}=P_{\text{CPA}}^{\infty }\;\cdot \;\text{exp}\left(-\frac{E_{a,\text{CPA}}}{\mathrm{RT}}\right)$; $P_{\text{Ions}}=P_{\text{Ions}}^{\infty }\;\cdot \;\text{exp}\left(-\frac{E_{a,\text{Ions}}}{\mathrm{RT}}\right)$, along with the inactive volume fraction $\upsilon_{B}$, membrane thickness h, elastic modulus of the cell membrane K, constant of membrane relaxation rate $k_{S}$, and membrane tension at resting $\sigma_{R}$.

**Figure 4:**
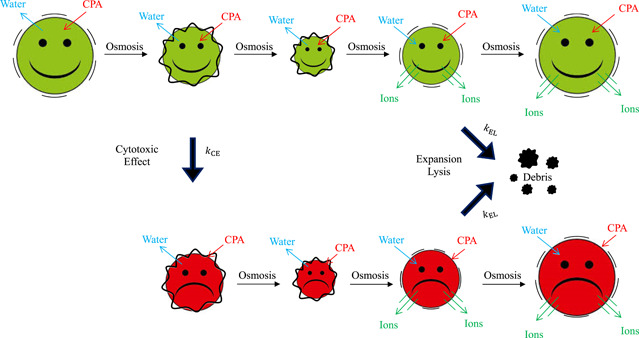
Schematic representation of cell system response to the additional stage of a toxic CPA like dimethyl sulfoxide, when the shrink‐swell dynamics occur according to the SAR model. Cell population is divided between viable (green) and nonviable (red) cells: Viable cells transform into nonviable ones following the reaction kinetics of cytotoxicity when a sufficient amount of intracellular CPA is accumulated; both sub‐populations decrease in number due to osmotic injury following the reaction kinetics of expansion lysis, that is, transforming into lost, undetected cells like debris, when the membrane is stretched too rapidly during swelling. CPA, Cryo‐Protectant Agent; SAR, surface area regulation.

In the present work the coupling of osmosis with cell mechanics and membrane SAR realized by the SAR model is used to define the kinetics of expansion lysis due to excessive osmotic swelling: following a first‐order reaction rate, viable as well as nonviable cells burst during swelling, when membrane tension increases too rapidly above a critical value. This is schematically depicted in Figure 4, where the stage of CPA addition (i.e., the contact phase) is represented by the well‐known shrink‐swell dynamics: initially, cell size decreases due to water outflow while CPA is accumulated in the cytoplasm, with cell membrane becoming slack and membrane tension lowering; thereafter, cell volume starts returning back towards its initial, isotonic value due to water inflow while CPA continues to enter the cells. This swelling causes the stretching of cell membrane that opens mechanosensitive channels (allowing ion exchange) and expansion lysis may occur. On the other hand, in Figure 4 cytotoxicity by DMSO is described as the kinetics of a simultaneous reaction: a first‐order reaction rate from viable (reactant, green) to nonviable (product, red) cells.

Therefore, according to this picture the cell population is divided into two subpopulations, that is, viable (V) and nonviable (NV) cells, as expressed by the following equation:

(1) $$N_{\text{TOT}}\left(t\right)=N_{V}\left(t\right)+N_{\text{NV}}\left(t\right)$$

where $N_{i}$ with i=TOT,V,NV represents the number of cells; nonviable cells refer to the measured sum of the necrotic and apoptotic cells reported in Figure 3b.

The two subpopulations of viable and nonviable cells ($N_{V}$ and $N_{\text{NV}}$) vary in time according to the reaction scheme depicted in Figure 4: when a sufficient intracellular concentration of the toxic and permeant DMSO is reached during osmotic excursions, viable cells are transformed in time to nonviable ones by following the reaction kinetics of the cytotoxic effect. This accounts for the decrease in cell viability experimentally measured by flow cytometry. For this reason, the number of viable and nonviable cells are expressed as a function of time (t) in Equation 1, with the first ones acting as the reactant which decreases in time being gradually transformed into the latter ones representing the reaction product. Actually, this reaction may take place during the contact phase (i.e., shrink‐swell dynamics during CPA addition) as well as during the subsequent stages when the cells are first pelleted by centrifugation, and finally suspended back to isotonic conditions (i.e., swell‐shrink dynamics during CPA removal), provided that a sufficient amount of DMSO is still present inside the cells.

Clearly, this transformation of a cell sub‐population into the other does not account for the temporal dependence of the total number of cells $N_{\text{TOT}}\left(t\right)$ appearing in Equation 1, which is exclusively due to the reaction representing expansion lysis shown in Figure 4 as well. According to this scheme, both viable and nonviable cells play the role of consumed reactants in the reaction representing osmotic injury, assuming that both subpopulations of cells share the same osmotic response as reported in Figure 4 for both green and red cells. This assumption is valid at the relatively low DMSO concentrations used in this study (cf. Table 1), when only a decrease in membrane thickness is expected to take place without any leakage effect according to Gurtovenko and Anwar (2007). In fact, this simplification of the model applies during the contact phase as well as during the subsequent removal phase, when the cells are suspended back to isotonic conditions: whenever an excessive swelling occurs, expansion lysis transforms viable and nonviable cells into lost, undetected cells (debris), thus mimicking the decrease of cell count experimentally measured by the Coulter counter.

Based on this picture, the following number balances in a closed reacting system may be written with the corresponding initial conditions:

(2) $$\frac{dN_{V}\left(t\right)}{\mathrm{dt}}=-\left(k_{\text{CE}}+k_{\text{EL}}\right)N_{V}\;\;\;@\;\;\;t=0\;\;\;N_{V}\left(t\right)=N_{V}^{0}=N_{\text{TOT}}^{0}R_{V}^{0}$$

(3) $$\frac{dN_{\text{NV}}\left(t\right)}{\mathrm{dt}}=k_{\text{CE}}N_{V}-k_{\text{EL}}N_{\text{NV}}\;\;\;@\;\;\;t=0\;\;\;N_{\text{NV}}\left(t\right)=N_{\text{NV}}^{0}=N_{\text{TOT}}^{0}\left(1-R_{V}^{0}\right)$$

where the kinetics of the reaction rates representing the cytotoxic effect and expansion lysis, namely $k_{\text{CE}}\;\cdot \;N_{V}$, and $k_{\text{EL}}\;\cdot \;N_{i}$ with i=V,NV respectively, is assumed to be first‐order with respect to the corresponding number of cells acting as reactants. The initial conditions are defined on the basis of the viability ratio $\left(R_{V}(t)=\frac{N_{V}(t)}{N_{\text{TOT}}(t)}\right)$ evaluated at t=0 ($R_{V}^{0}$).

According to Equations 2 and 3, while nonviable cells are produced by cytotoxicity and consumed by expansion lysis, viable cells are consumed by both reactions. Clearly, by summing up Equations 2 and 3 the balance on $N_{\text{TOT}}$ is obtained:

(4) $$\frac{dN_{\text{TOT}}(t)}{dt}=-k_{\text{EL}}\underset{N_{\text{TOT}}}{\underbrace{\left(N_{V}+N_{\text{NV}}\right)}\;}\;\;@\;\;\;t=0\;\;\;N_{\text{TOT}}(t)=N_{\text{TOT}}^{0}$$

confirming that, in the proposed model, only expansion lysis is seen as responsible for the decrease of the total number of cells.

Basically, according to the proposed model in combination with the experimental analysis carried out in this study, the hypothesis is that osmotic damage and CPA toxicity may be distinguished by using the total cell number as an indicator of osmotic damage, and the viable cell number as an indicator of toxicity, at least in the range of the operating conditions tested in this study and for the specific system DMSO/hMSCs. Thus, the possibilities that CPA toxicity leads to complete cell destruction by causing a decrease in total cell count, or that the osmotic effects alone lead to nonviable cells without complete cell destruction, are ruled out.

**Figure 5:**
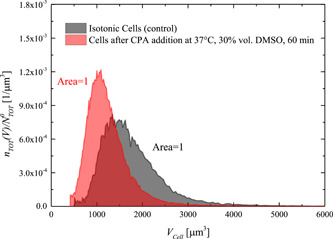
Normalized cell number density distribution measured by Coulter counter at the end of the contact phase at 37°C, 30 vol.% of DMSO, and 60 min, and for the initial isotonic cells. DMSO, dimethyl sulfoxide.

To justify these assumptions, it is worth noting that, according to (Gurtovenko & Anwar, 2007), only at very large DMSO concentrations (above ~25 mol%, much larger than the ones used in this study <8 mol%, cf. Table 1) the cells are expected to be completely destroyed by CPA toxicity thus causing a decrease of total cell count. This has been verified experimentally by measuring the size distribution of the cells right after the contact phase (before the CPA removal phase) at 30 vol.% DMSO, 37°C, and 60 min, that is, the worst‐case scenario for the cytotoxic effect among the runs performed in this study. Actually, at these operating conditions also the minimum shrinkage of cell volume is attained (among the runs performed in this study) during the shrink‐swell dynamics occurring in the DMSO addition phase. In Figure 5 the data measured by the Coulter counter under these operating conditions in terms of cell size distribution normalized with the initial total cell count are compared with the corresponding one measured for the initial, isotonic cells (i.e., control sample). As clearly shown, the area under the curve sums up to 1 in both cases, testifying that cytotoxicity itself and the shrinkage of cell volume are not able to cause a decrease in total cell count during the experiments performed in this study. On passing, it should be noted that data in Figure 5 show that at the end of DMSO addition (i.e., after the contact phase, when first shrinkage and then swelling already took place) the cells do not even return to the initial isotonic volume, but remain relatively smaller. This peculiar behavior can be interpreted by the SAR model where transmembrane exchange of ions between the cytoplasm and the suspending solution is allowed (i.e., intracellular ions may exit the cells during swelling, when cell membrane stretches and mechanosensitive channels open), whereas, according to the Kedem and Katchalsky or two‐parameter formalisms, the addition of a permeant CPA should always result in a cell volume larger than the initial isotonic one, depending on the extracellular concentration of the permeant CPA.

Regarding the other assumption that osmotic effects alone cannot lead to nonviable cells without complete cell destruction, the corresponding experimental check cannot be performed safely by means of the flow‐cytometric analysis adopted in this study. In fact, this technique requires the resuspension of the cells in a dye solution, thus causing secondary osmotic excursions in cell volume that may lead to misinterpretation of the results. Actually, this is the true reason why in this study cytotoxicity and osmotic injury have been evaluated together in a combined analysis only at the very beginning and at the end of the whole process, as schematically depicted in Figure 1. On the other hand, the assumption that osmotic effects alone cannot lead to nonviable cells is an implicit hypothesis made in the literature by many authors when analyzing the osmotic behavior of several cellular lineages: typically, in these works the volume excursions of initially isotonic cells suspended in hypo or hypertonic environments (obtained by diluting the buffer with pure water, or adding nontoxic and nonpermeant substances like sucrose) are measured during dynamic and equilibrium experiments. Then, these data are interpreted through the classic Kedem and Katchalsky or two‐parameter formalisms to determine water permeability, as well as by means of the Boyle Van't Hoff plot to evaluate the inactive cell volume. In all these works, even if relatively large volume excursions are actually achieved, typically the cells are assumed to respond to the imposed osmotic driving force by maintaining constant their osmotic parameters, that is, by remaining the same initially viable cells during the entire duration of the experiments, usually without any check on final viability.

### 3.1 Rate constant for cytotoxicity ($k_{\mathrm{CE}}$)

In the kinetics of the cytotoxic reaction shown in Figure 4, CPA is assumed to play the role of a homogenous catalyst whose intracellular concentration ($M_{\text{CPA}}^{\mathrm{INT}}$) varies in time according to the description of the osmotic response of the cells provided by the SAR model. Following the literature of homogeneous catalysis, for the reaction rate constant of cytotoxicity ($k_{\text{CE}}$) a generic power‐law dependence is assumed with respect to the intracellular concentration of the CPA (Chaudhari et al., 2001):

(5) $$k_{\text{CE}}=k_{\text{CE}}^{0}\;\text{exp}\left(-\frac{E_{a,\text{CE}}}{\mathrm{RT}}\right)\;{\left(M_{\text{CPA}}^{\mathrm{INT}}\right)}^{\alpha }$$

while the classic Arrhenius‐like dependence from system temperature is adopted (Benson et al., 2012; Davidson et al., 2015; Elmoazzen et al., 2007). As a consequence, $k_{\text{CE}}$ is not a true constant but varies with T and the temporal dependence of $M_{\text{CPA}}^{\mathrm{INT}}$ during the experimental runs performed in this study. The phenomenological character of Equation 5 is apparent: the order α is seen as a characteristic feature of the specific cell lineage‐CPA system at hand and its value may be estimated only through comparisons to experimental data, just like the pre‐exponential factor $k_{\text{CE}}^{0}$ and the activation energy $E_{a,\text{CE}}$. If both reaction order α and activation energy $E_{a,\text{CE}}$ are positive, the cytotoxic effect is expected to increase with temperature and CPA concentration.

### 3.2 Rate constant for expansion lysis ($k_{\mathrm{EL}}$)

The reaction rate constant $k_{\text{EL}}$ representing expansion lysis is defined on the basis of the SAR model where cell osmosis is coupled to membrane mechanics and SAR. In particular, in the SAR model, a cell under isotonic conditions is seen as an inflatable balloon whose surface is initially stretched at a resting tension (${\sigma =\sigma }_{R}$) representing a homeostatic condition. In response to a proper osmotic gradient, the cell inflates and an elastic stretching of the cell membrane ($\sigma >\sigma_{R}$) occurs during swelling. This stretching is only temporary though: it eventually vanishes when membrane SAR brings the tension back to its resting value to maintain cell homeostasis, through the exchange of surface area with membrane reservoirs.

According to this picture, the reaction rate constant of expansion lysis during swelling is assumed to be a function of the cell membrane tension above its resting value, that is, $k_{\text{EL}}(\Delta \sigma )$ with $\Delta \sigma =\left(\sigma -\sigma_{R}\right)>0$. Therefore, since a temporal variation of cell membrane tension in response to osmosis is accounted for by the SAR model, even $k_{\text{EL}}$ is not a true constant but varies with time.

Following this line of reasoning, expansion lysis is expected to occur if a critically high membrane tension $\sigma_{\mathrm{Break}}>\sigma_{R}$ is achieved during the osmotic swelling: when σ overcomes $\sigma_{\text{Break}}$ (i.e., when Δσ increases up to $\Delta \sigma_{\text{Break}}=\sigma_{\text{Break}}-\sigma_{R}>0$) the cells should burst losing their identity and transforming into debris. This way, from a mechanical perspective cell behavior during osmotic swelling is depicted as analogous to a classic brittle material in a tensile test: a linear, elastic response until the tensile strength is reached and breakage occurs.

To model expansion lysis as a kinetic phenomenon evolving in time, heterogeneity in the cell population during lysis must be introduced. This cannot be achieved by looking for differences among the cells since a single cell model is adopted in this study (all cells share the same volume), and viable as well as nonviable cells exhibit the same osmotic behavior, that is, the same Δσ temporal profile. A way to reach this goal is moving from a deterministic description of the occurrence of cell lysis event toward its statistical representation, by following the same approach adopted by the authors (Fadda et al., 2012a, 2012b) to model the mitotic rate in a growing cell population.

Basically, the model constraint of expansion lysis taking place exclusively when reaching $\Delta \sigma_{\text{Break}}$ is relaxed by adopting the following Weibull distribution

(6) $$f_{\mathrm{EL}}(∆\sigma )=\frac{K_{W}\;{\left(∆\sigma \right)}^{K_{W}-1}}{{\left(∆\sigma_{\text{Break}}\right)}^{K_{W}}}\;\text{exp}\left[-{\left(\frac{∆\sigma }{∆\sigma_{\text{Break}}}\right)}^{K_{W}}\right]$$

to represent the probability density function for the expansion lysis event. Here two adjustable parameters are introduced, namely, $K_{W}$ and $∆\sigma_{\text{Break}}$: depending on the value of the so‐called shape factor $K_{W}$, the probability of expansion lysis occurrence $f_{\mathrm{EL}}\left(∆\sigma \right)$ may show a monotonic decrease ($K_{W}<1$), or it may be characterized by a maximum ($K_{W}>1$), whose position is related to the specific value of the scale factor $∆\sigma_{\text{Break}}$. By increasing the shape factor $K_{W}$ above 1 the Weibull distribution narrows around a maximum located at $∆\sigma_{\text{Break}}$. This way, expansion lysis is depicted as a statistical event that may occur even at ∆σ lower or larger than $∆\sigma_{\text{Break}}$, but is maximum probability is at $∆\sigma_{\text{Break}}$.

According to (Hatzis et al., 1995; Koch & Schaecter, 1962), once the probability density function $f_{\mathrm{EL}}\left(∆\sigma \right)$ is specified, the corresponding transition rate $k_{\text{EL}}\left(∆\sigma \right)$ may be determined (and vice versa) by means of the following relationship:

(7) $$k_{\text{EL}}\left(∆\sigma \right)=\frac{d∆\sigma }{\mathrm{dt}}\;\gamma \left(∆\sigma \right)$$

where the rate of membrane tension variation ($\frac{d∆\sigma }{\mathrm{dt}}$) is calculated as the analytical derivative of Equation A.7 of the SAR model giving

(8) $$\frac{d∆\sigma }{\mathrm{dt}}=\left(\sigma +\frac{K}{2}\right)\left(\frac{2}{3V_{\text{Cell}}}\frac{dV_{\text{Cell}}}{\mathrm{dt}}-k_{S}∆\sigma \right)$$

and the so‐called transition function which is defined as $\gamma \left(∆\sigma \right)=\frac{f_{\mathrm{EL}}\left(∆\sigma \right)}{1-\int_{0}^{∆\sigma }f_{\mathrm{EL}\left({∆\sigma }^{\prime }\right)}{d∆\sigma }^{\prime }}$. In particular, for the case of the Weibull distribution used in Equation 6, if $K_{W}>1$ the following transition function is readily derived:

(9) $$\gamma \left(∆\sigma \right)=\frac{K_{W}\;{\left(∆\sigma \right)}^{K_{W}-1}}{{\left(∆\sigma_{\text{Break}}\right)}^{K_{W}}}$$

According to these equations, with an increasing shape factor (larger than 1) the Weibull distribution narrows around a maximum located at $∆\sigma_{\text{Break}}$, while the transition function γ(∆σ) increases monotonically toward infinitely large values. This means that $f_{\text{EL}}\left(∆\sigma \right)$ is maximum at ∆σ close to $∆\sigma_{\text{Break}}$, while, at any given $\frac{d∆\sigma }{\mathrm{dt}}$, the rate of expansion lysis $k_{\text{EL}}\left(∆\sigma \right)$ accelerates as much as membrane tension ∆σ overcomes $∆\sigma_{\text{Break}}$. In other words, expansion lysis is seen as a statistical event where cells are able to reach a membrane tension greater than $\sigma_{\text{Break}}$ without lysis, but its probability increases so much that it will soon occur shortly after.

It is worth noting that, $k_{\text{EL}}\left(∆\sigma \right)$ is determined through Equation 7 only if $\frac{d∆\sigma }{\mathrm{dt}}>0$ and ∆σ>0, otherwise it is set equal to zero. Thus, a conditional osmotic injury is hypothesized: expansion lysis occurs only if the cell membrane is already stretched (i.e., during swelling) and its tension is increasing in time, namely ∆σ>0 and $\frac{d∆\sigma }{\mathrm{dt}}>0$, respectively. On the contrary, expansion lysis does not occur if cell membrane is at resting conditions or loose like during osmotic shrinkage (i.e., ∆σ≤0), or when an already stretched membrane is actually relaxing (i.e., $\frac{d∆\sigma }{\mathrm{dt}}\leq 0$ with ∆σ>0).

**Figure 6:**
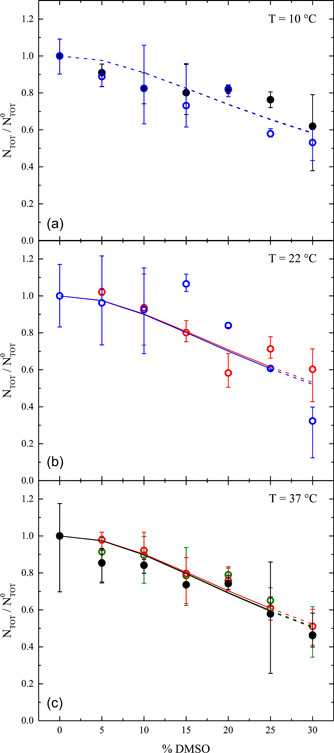
Normalized cell count measured by Coulter counter at the end of the removal phase as a function of vol.% of DMSO used during the contact phase: varying the temperature during the contact phase (a) 10°C; (b) 22°C; (c) 37°C, and contact times 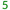, 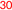, 60, 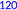 min. Symbol represents measured data, the solid line represents model fitting, and dashed line represents model prediction. DMSO, dimethyl sulfoxide.

**Figure 7:**
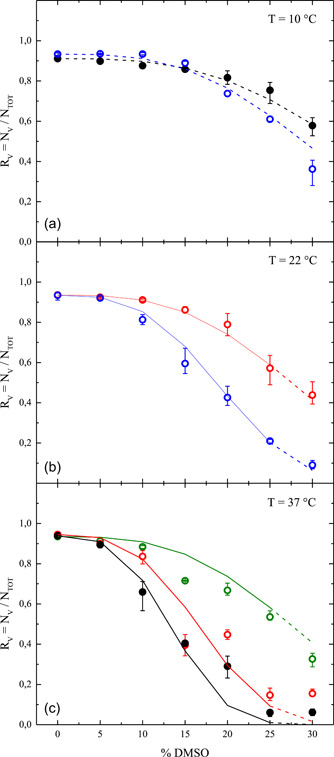
Viability ratio measured by flow‐cytometer at the end of the removal phase as a function of vol.% of DMSO used during the contact phase: Varying the temperature during the contact phase (a) 10°C; (b) 22°C; (c) 37°C, and contact times 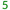, 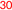, 60, 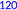 min. Symbol represents measured data, solid line represents model fitting, dashed line represents model prediction. DMSO, dimethyl sulfoxide.

**Table 2:** Parameter values of the reaction rates representing the cytotoxic effect and expansion lysis

Parameter	Value (95% confidence interval)	Unit
$E_{a,\text{CE}}$	92,816.592 (±1)	[J mol^−1^]
$K_{W}$	2.5456 (±10.0)	[−]
$k_{\text{CE}}^{0}$	61.785 (±4.1 × 10^−4^)	$\left[L^{\alpha }\;s^{-1}\;{\text{mOsm}}^{-\alpha }\right]$
α	3.0822 (±0.0)	[−]
$\sigma_{\text{Break}}$	10,985.156 (±30,880.344)	[Pa]

## 4 RESULTS AND DISCUSSION

In this study, the SAR model is used to describe the osmotic response of the cells during the experimental runs, where expansion lysis, as well as cytotoxicity, may take place. To this aim, the equations of the SAR model reported in the Appendix section are coupled with Equations (1), (2), (3), (4), (5), (6), (7), (8), (9). More specifically, the SAR model is used to simulate $M_{\text{CPA}}^{\mathrm{INT}}(t)$, ∆σ(t), and $\frac{d∆\sigma }{\mathrm{dt}}$ appearing in Equations (5), (6), (7), (8), (9) to determine the kinetic constants $k_{\text{CE}}$ and $k_{\text{EL}}$ of the reaction rates representing the cytotoxic effect and expansion lysis, correspondingly. This system of Equations is numerically solved by means of a home‐made Fortran program using a shareware routine (LSODE) for the integration of an Initial Value Problem for a system of Ordinary Differential Equations.

Basically, in the model proposed in this work five parameters are introduced to describe cytotoxicity and expansion lysis, namely: $k_{\text{CE}}^{0}$, $E_{a,\text{CE}}$, and α (for the reaction rate of the cytotoxic effect), $K_{W}$ and $\sigma_{\text{Break}}$ (for the reaction rate of expansion lysis). The values of these five parameters are determined through regression analysis (minimization of squared errors) by keeping constant the osmotic parameters of the SAR model reported in Table A3: in particular, only the data at 22°C and 37°C (up to 25 vol.% of DMSO for any contact time) among the experimental runs listed in Table 1 are best‐fitted through the comparisons to model results. The data from the experimental runs at 10°C (for any vol.% of DMSO and contact time) and those at 22°C and 37°C (only at 30 vol% of DMSO but for any contact time) are used for model validation, through prediction of system behavior, that is, without adjusting any parameter.

In Figures 6 and 7 the comparisons between data and model results are shown, while the values of the adjustable parameters obtained from the regression analysis are reported in Table 2, along with the corresponding 95% confidence intervals (CI). Taking advantage of the fact that the decrease of total cell count depends only on expansion lysis (cf. Equation 4), the five adjustable parameters have been determined in a sequential fashion: first, the two parameters $K_{W}$ and $\sigma_{\text{Break}}$ of expansion lysis have been adjusted to fit only cell count data (cf. Figure 6); then, the remaining three parameters $k_{\text{CE}}^{0}$, $E_{a,\text{CE}}$, and α of the cytotoxic effect have been determined through regression of the cell viability data (cf. Figure 7), by keeping constant the values of the two parameters $K_{W}$ and $\sigma_{\text{Break}}$ previously obtained.

In particular, in Figure 6 the cell count (normalized with the corresponding initial value) measured by the Coulter counter at the end of the removal phase is reported as a function of the vol.% of DMSO used during the contact phase, for every experimental run in Table 1. Analogously, in Figure 7 the viability ratio measured by the flow‐cytometer at the end of the removal phase is shown as a function of vol.% of DMSO used during the contact phase. For the sake of comparison, separated data are shown for the three temperature levels used during the contact phase, while the varying contact time is accounted for by using different colors, that is, 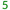, 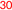, 60, and 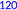 min, for data (closed circle) as well as theoretical results (solid line for model fitting and dashed line for model prediction).

Both data of cell count in Figure 6 and viability ratio in Figure 7 are clearly shown to decrease as DMSO concentration increases. However, whereas data of viability ratio are shown in Figure 7 to vary also with temperature and contact time in a consistent fashion, only scattered measurements of cell count are obtained at varying temperature and contact time as reported in Figure 6, especially at 10°C and 22°C. In fact, the measurements of cell count obtained through the Coulter counter are characterized by a much greater level of variability than those ones of viability ratio given by the flow‐cytometer, as testified by the error bars shown in Figure 6. Moreover, it is worth noting that a fraction of nonviable cells (about 10%) is always initially present in the sample given that the data of viability ratio shown in Figure 7 always starts from a value lower than 1.

As a consequence, the comparisons with model results on cell count in Figure 6 are less satisfactory than those on viability in Figure 7. In particular, model fitting and predictions for cell count almost overlap showing a clear dependence on DMSO concentration, regardless of temperature and contact time. This dependence is apparently double linear: a slow decrease at DMSO vol.% lower than 5−10, with an increased slope at higher concentration levels. On the contrary, cell viability in Figure 6 shows a sigmoidal decrease with respect to DMSO concentration, with a clear detrimental effect when increasing temperature and contact time. Overall, model fittings and predictions may be considered satisfactory, especially if compared to other similar studies available in the literature where no prediction was provided for model validation and osmotic injury was neglected (Benson et al., 2012; Elmoazzen et al., 2007; Hunt et al., 2003a, 2003b; Wang et al., 2007) or avoided by working within the range delimited by preset OTLs (Davidson et al., 2015).

Regarding the specific values of the parameters reported in Table 2, for the kinetics of cytotoxicity an almost doubled reaction order α (i.e., ~3.1 vs. 1.6) and activation energy $E_{a,\text{CE}}$ (i.e., ~93 kJ mol^−1^ vs. 56 kJ mol^−1^) are found in this study in comparison with those reported in the literature for other cell lines in contact with other CPAs (Benson et al., 2012; Davidson et al., 2015). On the other hand, a remarkable similarity is obtained for expansion lysis parameters given that (Wolfe et al., 1986) reported a maximum surface tension tolerated by protoplasts during an osmotic expansion (4 mN m^−1^) comparable to the one obtained in this study for hMSCs ($\sigma_{\text{Break}}\;\cdot \;h$ = 5.55 mN m^−1^), even if with different surface tension at resting/homeostatic condition (0.1 mN m^−1^ vs. $\sigma_{R}\;\cdot \;h$ = 0.4 mN m^−1^) and different elastic modulus (200 mN m^−1^ vs. K·h = 16.5 mN m^−1^).

However, whereas the 95% CI shown in Table 2 reveal that the values assigned to the parameters related to cytotoxicity are extremely reliable, conversely the values assigned to the parameters related to expansion lysis are uncertain. These opposite results are mainly due to the larger variability of cell count measurements through the Coulter counter than those ones of viability ratio given by the flow‐cytometer. In addition, the adoption of the Weibull distribution in Equation 6 may play a role in the excessively large 95% CI for the parameters related to expansion lysis. In fact, the shape of the Weibull distribution strongly depends on the value of $K_{W}$ being lower or larger than 1, shifting from a monotonically decreasing function to a bell‐shaped function, thus completely changing the condition of the lysing cells. Presumably, by choosing another distribution to represent the probability density function for the expansion lysis event (for instance, a log‐normal distribution with three adjustable parameters) the 95% CI for the two parameters related to expansion lysis will be narrowed down.

**Figure 8:**
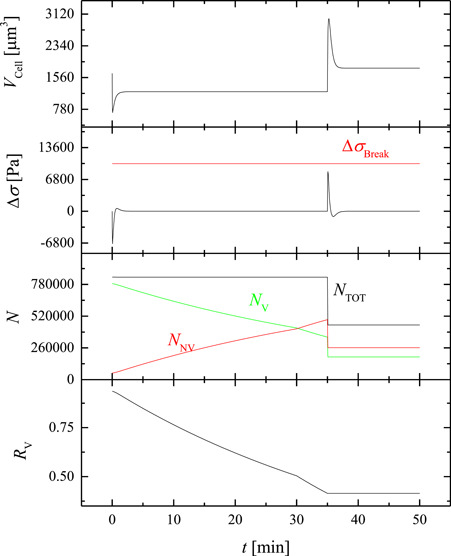
Temporal profiles of cell volume ($V_{Cell}$), cell membrane tension above resting condition (∆σ), total cell count ($N_{\text{TOT}}$), number of viable ($N_{V}$) and nonviable ($N_{\mathrm{NV}}$) cells, and viability ratio ($R_{V}=\frac{N_{V}}{N_{\text{TOT}}}$) for the experimental run of a contact phase at 22°C, 30 vol.% DMSO, and 30 min as contact time. DMSO, dimethyl sulfoxide.

To better understand system behavior, the simulated temporal profiles of some key variables of the proposed model are shown in Figure 8 for the experimental run of a contact phase at 22°C, 30 vol.% DMSO, and a contact time of 30 min taken as representative. In particular, the transient cell volume ($V_{\text{Cell}}$) is reported for this case along with the corresponding cell membrane tension above resting condition (∆σ), total cell count ($N_{\text{TOT}}$), number of viable ($N_{V}$) and nonviable ($N_{\text{NV}}$) cells, and viability ratio ($R_{V}=\frac{N_{V}}{N_{\text{TOT}}}$). It is apparent that, during the contact phase (i.e., for t< 30 min) cells show the classic shrink‐swell dynamics due to a membrane permeability to DMSO lower than water, followed by 5 min of centrifugation and the swell‐shrink dynamics during the removal phase (i.e., for t> 35 min). The cytotoxic effect is basically confined in the contact phase: this is demonstrated by the continuous decrease of the number of viable cells $N_{V}$ transformed into nonviable cells $N_{\text{NV}}$ that grow in number, while $N_{\text{TOT}}$ does not vary until the removal phase starts at 35 min. As a consequence, the viability ratio $R_{V}$ continuously declines in the first 30 min, with an even increased slope in the following 5 min when cytotoxicity by intracellular DMSO continues during centrifugation at a higher temperature (room temperature, 27°C). Later, when the removal phase begins at t= 35 min, the cytotoxic effect stops as the intracellular concentration of DMSO rapidly diminishes, and expansion lysis occurs abruptly: this is demonstrated by the drop of $N_{\text{TOT}}$ as well as $N_{V}$ and $N_{\text{NV}}$ while viability ratio remains constantly equal to the value reached at the end of centrifugation. Basically from Figure 8, it is apparent that for hMSCs in contact with DMSO cytotoxicity and expansion lysis do not occur simultaneously, even if the proposed model does not exclude this possibility: more specifically, cytotoxicity takes place only when the intracellular concentration of DMSO is relatively high during the contact and centrifugation phases; on the other hand, expansion lysis is confined to the osmotic swelling during the removal phase when the cell membrane is significantly stretched above its resting condition as shown by the positive values reached by ∆σ. Actually, a small increase of ∆σ>0 occurs even during the contact phase, when the cells swell back after the initial cell shrinkage: but this is a temporary and negligible increase for cell membrane stretching that is not capable to damage the cells.

This temporal separation between cytotoxicity and expansion lysis described by the proposed model explains the reason why the uncertainty about the values assigned to the lysis parameters fail to affect the reliability of the values of the cytotoxicity‐related parameters (cf. 95% CI in Table 2), despite the regression of the latter ones was performed in sequence after that of the former ones: when cell viability is reduced by cytotoxicity during CPA addition the total cell count $N_{\text{TOT}}$ does not really drop and remains equal to $N_{\mathrm{TOT}}^{0}$ (i.e., $R_{V}=\frac{N_{V}}{N_{\text{TOT}}}=\frac{N_{V}}{N_{\mathrm{TOT}}^{0}}$) until the lethal swelling occurs during the subsequent CPA removal phase.

Moreover, it is apparent that the cytotoxic effect is definitely slower than osmosis (which is concluded within 5 min), but it's much slower than expansion lysis which is almost instantaneous in comparison.

**Figure 9:**
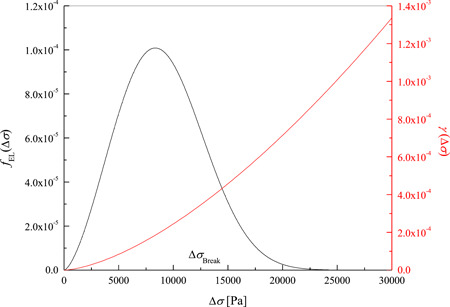
Weibull distribution $f_{\text{EL}}\left(∆\sigma \right)$ of Equation 6 and transition function γ(∆σ) of Equation 9 corresponding to the parameter values of $K_{W}$ and $\sigma_{\text{Break}}$ determined for human Mesenchymal Stem Cells as reported in Table 2.

It is worth noting that, as shown in Figure 8 the positive ∆σ never reaches $∆\sigma_{\text{Break}}$ actually, even when expansion lysis occurs during the swelling of the removal phase. To explain this, Figure 9 shows the plot of the Weibull distribution $f_{\text{EL}}\left(∆\sigma \right)$ and transition function γ(∆σ) corresponding to the values of $K_{W}$ and $\sigma_{\text{Break}}$ reported in Table 2. Here it is shown that in the simulations of the proposed model the Weibull distribution is a log–normal function (i.e., bell‐shaped with positive skewness), with a single mode slightly smaller than $∆\sigma_{\text{Break}}$. This result is due to a shape factor $K_{W}$ larger than 1 but not large enough for the coincidence of mode and mean of the distribution at $∆\sigma =∆\sigma_{\text{Break}}$. This means that the probability for the occurrence of expansion lysis is higher at ∆σ smaller than $∆\sigma_{\text{Break}}$ as results from Figure 8, while the corresponding transition function γ(∆σ) shown in Figure 9 does not increase so much past $∆\sigma_{\text{Break}}$. This means that for the rate of expansion lysis in Equation 7 the term representing how fast membrane tension varies with time $\frac{d∆\sigma }{\mathrm{dt}}$ plays a significant role: during the osmotic swelling, at the beginning of the removal phase, a very rapid increase of membrane stretching occurs leading to cell burst, while a negligible osmotic injury takes place during the slower, secondary swelling of the contact phase. This different behavior of the system is due to water permeability through the cell membrane which is much larger than DMSO permeability: the latter rules the slow swelling rate during the contact phase, while the first one determines a very fast cell volume expansion at the beginning of the removal phase.

## 5 CONCLUSIONS

In this study, a combined analysis of the osmotic injury and cytotoxic effect when hMSCs from UCB are contacted with DMSO is carried out from the experimental and theoretical perspectives. A novel mathematical model is developed and proposed to interpret data, without the need to set in advance the cell OTLs, as traditionally done in the cryopreservation literature. The model is based on the adoption of the SAR model recently proposed by the authors to describe the nonperfect osmotic behavior of hMSCs, where osmosis is coupled with cell mechanics and membrane area regulation. Comparisons between experimental data and theoretical simulations are provided, first by performing a nonlinear regression analysis to evaluate unknown model parameters, then by checking its reliability to predict system behavior measured at operating conditions different from those used during the best‐fit procedure.

Based on our model simulations it is found that the cytotoxic effect of DMSO on hMSCs is slower than osmosis and expansion lysis. Moreover, the cytotoxic effect is confined to CPA addition, while expansion lysis occurs during CPA removal. Therefore, for this cells‐CPA system a considerably reduced cytotoxic effect should be achieved if CPA addition is carried out at low temperatures, and by limiting the contact time to the one strictly necessary for osmotic equilibration. Regarding this, the temperature of the system should be optimized by balancing between a reduced cytotoxic effect and a longer osmotic equilibration as the temperature is reduced.

This means that a one‐step strategy may be safely realized for the contact phase between DMSO and hMSCs, that is, the whole DMSO may be abruptly added to the extracellular solution to shorten the contact time. On the contrary, since expansion lysis during the removal phase is very fast, rapid membrane stretching should be carefully avoided by adopting a multi‐step strategy, that is, by sequentially suspending the cells in a series of washing solutions with lowering osmotic tonicity. In this case too, system temperature should be optimized by balancing between reduced osmotic lysis and a longer osmotic equilibration as the temperature is lowered. The proposed model can be adopted to design and optimize this strategy, by automatically taking into account expansion lysis without the need to pre‐set fixed OTLs.

## NOMENCLATURE

$k_{\text{CE}}$
: cytotoxic effect reaction rate [s^−1^]
$k_{\text{CE}}^{0}$
: cytotoxic effect reaction rate parameter [$L^{\alpha }$ s^−1^ ${\text{mOsm}}^{-\alpha }$]
$k_{\text{EL}}$
: expansion lysis reaction rate [s^−1^]
$E_{a}$
: activation energy [J mol^−1^]
$E_{a,\text{CE}}$
: cytotoxic effect reaction activation energy [J mol^−1^]
$f_{\text{EL}}$
: expansion lysis Weibull distribution [Pa^−1^]
h
: cell membrane thickness [µm]
K
: young modulus—elastic constant [Pa]
$K_{W}$
: exponent of the Weibull distribution [‐]
$k_{S}$
: SAR rate constant [Pa^−1^ s^−1^]
$L_{P}$
: water permeability [µm Pa^−1^ s^−1^]
M
: osmolality [mOsm L‐1]
n: cell number density distribution [µm^−3^]
N
: number [‐]
P
: hydrostatic pressure [Pa]
$P_{\text{CPA}}$
: CPA permeability [µm s^−1^]
$P_{\text{Ions}}$
: ions permeability [µm L s^−1^ mOsm^−1^]
R
: universal gas constant [J mol^−1^ K^−1^]
r
: cell radius [µm]
$R_{V}$
: viability ratio [−]
$S_{\text{Sph}}$
: cell surface [µm^2^]
$S_{\text{Ref}}$
: cell reference surface [µm^2^]
T
: temperature [K]
t
: time [s]
$V_{B}$
: osmotic inactive volume fraction [µm^3^]
$V_{\text{Cell}}$
: mean cell volume [µm^3^]
α
: cytotoxic effect reaction rate parameter [−]
γ
: transition function [Pa^−1^]
Π
: osmotic pressure [Pa]
σ
: cell membrane tension [Pa]
$\tilde{\upsilon }$
: molar volume [m^3^ mol^−1^]
φ
: dissociation factor [−]

**SUPERSCRIPTS**
${\;}^{\text{EXT}}$
: referred to the extracellular solution
${\;}^{\mathrm{INT}}$
: referred to the intracellular solution
${\;}^{0}$
: referred to the initial time
${\;}^{\infty }$
: referred to infinite temperature

**SUBSCRIPTS**
${\;}_{\text{CPA}}$
: referred to the CPA
${\;}_{\text{Ions}}$
: referred to the Ions
${\;}_{\text{Break}}$
: referred to the critical tension
${\;}_{\text{Sucrose}}$
: referred to the nonpermeant sucrose
${\;}_{\text{NV}}$
: nonviable cells
${\;}_{R}$
: referred to the resting condition
${\;}_{\text{TOT}}$
: total cells
${\;}_{V}$
: viable cells
${\;}_{W}$
: referred to the water

## AUTHOR CONTRIBUTIONS

*Conceptualization and project administration*: Alberto Cincotti. *Data curation and formal analysis*: Gabriele Traversari. *Funding acquisition*: Santiago Aparicio and Alberto Cincotti. *Investigation and methodology*: Gabriele Traversari and Alberto Cincotti. *Supervision*: Santiago Aparicio, Francesco Delogu, and Alberto Cincotti. *Writing original draft, writing review, and editing*: Gabriele Traversari, Santiago Aparicio, Francesco Delogu, and Alberto Cincotti.

## 1

In the surface area regulation (SAR) model the description of cell osmosis is coupled with cell mechanics and cell membrane SAR. The model improves the two‐parameter formalism typically adopted in cryopreservation literature by allowing the conditional transmembrane permeation of ions/salt, through the temporary opening of mechanosensitive (MS) channels whenever membrane stretching occurs: this way cells can reach an equilibrium volume different from the initial one, when isotonic conditions are re‐established after contacting with impermeant or permeant solutes, such as sucrose or a cryoprotectant agent like dimethyl sulfoxide, respectively. In the classic two‐parameter and Kedem−Katchalsky formalisms, on the other hand, cells always return back to the isotonic volume showing a perfect osmometer behavior.

The equations are collectively reported in Tables A1, A2, with Ordinary Differential Equations conveniently separated by auxiliary Algebraic Equations. A thorough discussion of the equations of the SAR model with its development is available in the literature (Casula et al., 2019) and it is not repeated in this study, where only the following brief description is provided. Basically, in the SAR model a cell under isotonic conditions is seen as an inflatable balloon whose surface $S_{\text{Sph}}$ is initially stretched from $S_{\text{Ref}}$ at a resting tension $\sigma_{R}$ representing a homeostatic condition, as expressed by the initial condition of Equation A.4. In response to an osmotic gradient ΔM (defined in Equations A.11−A.16) a cell inflates or deflates through the exchange with the extracellular compartment of water, Cryo‐Protectant Agent, and ions (at the rates expressed in Equations A.1−A.3) thus changing its spherical volume $V_{\text{Cell}}$ and membrane surface area $S_{\text{Sph}}$ (determined by means of Equation A.5 and A.9, respectively). As a consequence, the ratio $\frac{S_{\text{Sph}}}{S_{\text{Ref}}}$ varies from its value at resting condition ($1+\frac{2\;\sigma_{R}}{K}$) in a proportional fashion with cell membrane tension σ, that is, an elastic response from a mechanical perspective, with K representing the nondimensional elastic modulus defined in Equation A.8. This variation has two consequences: according to the Laplace law (i.e., Equation A.6) a counter‐gradient of hydrostatic pressure ∆P always opposing the osmotic driving force ΔM in the water exchange rate emerges (note the opposite signs of the two driving forces in Equation A.1), while MS channels open allowing ion exchange (inward or outward depending on their gradient) if the membrane is stretched (see Equation A.17), that is, if $\sigma >\sigma_{R}$ or $\left(\frac{S_{\text{Sph}}}{S_{\text{Ref}}}\right)>\left(1+\frac{2\;\sigma_{R}}{K}\right)$. On the other hand, the variation of membrane tension with respect to the resting condition is only temporary due to membrane relaxation governed by Equation A.4: it eventually vanishes through the exchange of surface area with membrane reservoirs, which brings the membrane tension back to its resting value to maintain cell homeostasis.

**Table A1:** ODEs of the SAR model (Casula et al., 2019; Traversari & Cincotti, 2021)

Equation	Initial condition	Number
$\frac{dV_{W}}{\mathrm{dt}}=-L_{P}S_{\text{Sph}}\left(∆P-∆\Pi \right)$	$V_{W}(0)=V_{W}^{0}=\left(\;V_{\text{Cell}}^{0}-V_{\text{Ions}}^{0}-V_{B}\right)\;\;@\;\;t=0$	(A.1)
$\frac{dV_{\text{CPA}}}{\mathrm{dt}}=-{\tilde{\upsilon }}_{\text{CPA}}P_{\text{CPA}}S_{\text{Sph}}{\Delta M}_{\text{CPA}}$	$V_{\text{CPA}}(0)=V_{\text{CPA}}^{0}=0\;\;@\;\;t=0$	(A.2)
$\frac{dV_{\text{Ions}}}{\mathrm{dt}}=-P_{\text{Ions}}S_{\text{Sph}}{\Delta M}_{\text{Ions}}$	$V_{\text{Ions}}(0)=V_{\text{Ions}}^{0}=\frac{\left(V_{\text{Cell}}^{0}-V_{B}\right)}{1+\frac{\varphi }{{\tilde{\upsilon }}_{\text{Ions}}M^{0}}}\;\;@\;\;t=0$	(A.3)
$\frac{dS_{\text{Ref}}}{\mathrm{dt}}=k_{S}S_{\text{Ref}}∆\sigma$	$S_{\text{Ref}}(0)=S_{\text{Ref}}^{0}=\frac{S_{\text{Sph}}^{0}}{1+\frac{2\sigma_{R}}{K}}\;\;@\;\;t=0$	(A.4)

Abbreviations: ODEs, Ordinary Differential Equations; SAR, surface area regulation.

**Table A2:** AEs of the SAR model (Casula et al., 2019; Traversari & Cincotti, 2021)

Equation	Number
$V_{\text{Cell}}=V_{B}+V_{\text{Ions}}+V_{W}+V_{\text{CPA}}$	(A.5)
$\Delta P=P^{\text{INT}}-P^{\text{EXT}}=\frac{2h∆\sigma }{r}$	(A.6)
$\Delta \sigma =\sigma -\sigma_{R}$	(A.7)
$\sigma =\frac{K}{2}\left(\frac{S_{\text{Sph}}}{S_{\text{Ref}}}-1\right)$	(A.8)
$S_{\text{Sph}}=4\pi {\left(\frac{3\;V_{\text{Cell}}}{4\pi }\right)}^{\frac{2}{3}}$	(A.9)
$\Delta \Pi =\mathrm{RT}\Delta M=\mathrm{RT}\left(M^{\text{INT}}-M^{\text{EXT}}\right)$	(A.10)
$M^{\mathrm{INT}}=M_{\text{Ions}}^{\mathrm{INT}}+M_{\text{CPA}}^{\mathrm{INT}}$	(A.11)
$M_{\text{Ions}}^{\mathrm{INT}}=\frac{\varphi V_{\text{Ions}}}{{\tilde{\upsilon }}_{\text{Ions}}V_{W}}$	(A.12)
$M_{\text{CPA}}^{\mathrm{INT}}=\frac{V_{\text{CPA}}}{{\tilde{\upsilon }}_{\text{CPA}}V_{W}}$	(A.13)
$M^{\text{EXT}}=\left(M_{\text{Ions}}^{\text{EXT}}+M_{\text{Sucrose}}^{\text{EXT}}+M_{\text{CPA}}^{\text{EXT}}\right)$	(A.14)
$\Delta M_{\text{CPA}}=M_{\text{CPA}}^{\mathrm{INT}}-M_{\text{CPA}}^{\text{EXT}}$	(A.15)
$\Delta M_{\text{Ions}}=M_{\text{Ions}}^{\mathrm{INT}}-M_{\text{Ions}}^{\text{EXT}}$	(A.16)
$P_{\text{Ions}}=\left\{\begin{array}{l} 0\;\;\;\;\;\;\;\;\Delta \sigma \leq 0\\ P_{\text{Ions}}\;\;\;\Delta \sigma >0 \end{array}\right.$	(A.17)

Abbreviations: AEs, Algebraic Equations; SAR, surface area regulation.

**Table A3:** Parameter values of the SAR model (Casula et al., 2019; Traversari & Cincotti, 2021)

Parameter	Value	Unit
$E_{a,\text{CPA}}$	72,570	[J mol^−1^]
$E_{a,\text{Ions}}$	22,150	[J mol^−1^]
$E_{a,W}$	50,000	[J mol^−1^]
h	0.5	[µm]
K	33,000	[Pa]
$k_{S}$	3.7 × 10^‐6^	[Pa^−1^ s^−1^]
$L_{P}^{\infty }$	64.2	[µm Pa^−1^ s^−1^]
$P_{\text{CPA}}^{\infty }$	1.268 × 10^12^	[µm s^−1^]
$P_{\text{Ions}}^{\infty }$	4.47 × 10^‐3^	[µm L s^−1^ mOsm^−1^]
R	8.314472	[J mol^−1^ K^−1^]
$\sigma_{R}$	826	[Pa]
$V_{\text{Cell}}^{0}$	1800	[µm^3^]
$\upsilon_{B}=\frac{V_{B}}{V_{\text{Cell}}^{0}}$	0.2	[−]
${\tilde{\upsilon }}_{\mathrm{CPA}}$	7.1 × 10^−5^	[m^3^ mol^−1^]
${\tilde{\upsilon }}_{\text{Ions}}$	2.7 × 10^−5^	[m^3^ mol^−1^]
φ	2	[−]

Abbreviation: SAR, surface area regulation.
